# Distant Metastases in Patients with Intrahepatic Cholangiocarcinoma: Does Location Matter? A Retrospective Analysis of 370 Patients

**DOI:** 10.1155/2020/7195373

**Published:** 2020-10-10

**Authors:** Felix Hahn, Lukas Müller, Aline Mähringer-Kunz, Yasemin Tanyildizi, Daniel Pinto dos Santos, Christoph Düber, Peter R. Galle, Arndt Weinmann, Roman Kloeckner

**Affiliations:** ^1^Department of Diagnostic and Interventional Radiology, University Medical Center of the Johannes Gutenberg-University Mainz, Mainz, Germany; ^2^Department of Neuroradiology, University Medical Center of the Johannes Gutenberg-University Mainz, Mainz, Germany; ^3^Department of Radiology, University Hospital of Cologne, Cologne, Germany; ^4^Department of Internal Medicine, University Medical Center of the Johannes Gutenberg-University Mainz, Mainz, Germany; ^5^Clinical Registry Unit (CRU), University Medical Center of the Johannes Gutenberg-University Mainz, Mainz, Germany

## Abstract

**Background:**

Intrahepatic cholangiocarcinoma (ICC) is an aggressive tumor entity, and distant metastases are common. However, studies investigating patterns and clinical relevance of distant metastases are rare. Therefore, we aimed to analyze occurrence, location, and prognostic impact of distant metastases on overall survival (OS).

**Methods:**

Between 1997 and 2018, 417 patients with ICC were treated at our tertiary care center. Distant metastases and intrahepatic tumor burden were retrospectively evaluated in a longitudinal approach using volumetric assessment of cross-sectional imaging studies and all available medical/histopathological reports.

**Results:**

Finally, 370 patients with histopathologically confirmed ICC were included. Of these, 186 showed distant metastases, either initially (*n* = 59) or during follow-up (*n* = 127). The most common metastatic sites were the lung (*n* = 105), peritoneum (*n* = 81), and bone (*n* = 50). After detection of lung metastases, the residual median OS was 5.3 months; followed by peritoneal metastases, 4.5 months, and bone metastases, 4.4 months (*P*=0.17). At the time of first metastatic occurrence, residual OS according to intrahepatic tumor burden of <25%, 25–50%, and >50% was 6.5 months, 4.9 months, and 1.2 months, respectively (*P* < 0.001). In multivariate hazard regression, hepatic tumor burden, liver function, and subsequent treatment were significant predictors of survival.

**Conclusions:**

During the disease course, every second patient developed extrahepatic metastases. While the presence of distant metastases was associated with poor patient outcomes, there was no significant difference between metastatic sites. However, hepatic tumor burden was the life-limiting risk factor in a majority of patients at the time of distant metastatic disease.

## 1. Introduction

Intrahepatic cholangiocarcinoma (ICC) is the second most common primary liver malignancy after hepatocellular carcinoma (HCC). Incidence in Western countries is estimated to be approximately 0.4–2.0/100,000 and has considerably increased since the last three decades [1–4].

Affected patients are often asymptomatic in the early stages; thus at diagnosis, the tumor is often already at an advanced stage [5]. Nonresectable patients have repeatedly been identified as having a poorer prognosis than patients undergoing surgery [6, 7]. However, even if resection is possible, tumor recurrence has been reported in about 65% of the patients [8].

Regarding systemic chemotherapy, the combination of gemcitabine and cisplatin has been widely administered as first-line chemotherapy since the publication of the multicenter UK-ABC 02 study in 2010 [7]. Even though systemic chemotherapy is the mainstay of treatment, intra-arterial therapies such as transarterial chemoembolization (TACE) or selective internal radiation therapy (SIRT) have also been established since the last decade as treatment options for selected patients [9]. However, prognosis remains poor [10].

As resection is the only curative treatment option, the presence of distant metastases impedes curative-intent resections [11]. Distant metastases are incorporated into the prognostic Wang nomogram [12]; also, the discriminator between stage III and stage IV in the current 8^th^ edition of the UICC staging system is the presence of distant metastases [13]. In a recent study performed by our group, distant metastases were independent predictors of poor survival as well [14].

Based on the Surveillance, Epidemiology, and End Results (SEER) database, Wu et al. [15] investigated patterns of distant extrahepatic metastases in primary liver cancer, both HCC and ICC. However, the authors limited their investigation to patients with distant metastases at the time of diagnosis only. Moreover, survival analyses were presented for pooled HCC and ICC patients, with ICC patients accounting for only 11% of the study population.

To the best of our knowledge, no study has focused on the longitudinal investigation of metastatic occurrence and the impact of different sites of metastatic spread for patients with ICC. Therefore, the aim of this study was to analyze occurrence, location, and prognostic impact of distant metastases on overall survival (OS) during the course of disease.

## 2. Materials and Methods

Between January 1997 and January 2018, 417 patients with histopathologically confirmed ICC were treated at our tertiary care center. These patients were retrospectively identified from a dedicated, prospectively populated clinical database. Data from follow-up visits were extracted from the hospital and radiology information systems. Death dates were queried at the appropriate resident's registration offices. Follow-up was ended on December 31, 2018. In case of loss to follow-up, patients were censored at the date of last contact. The study was approved by the responsible ethics committee for the retrospective analysis of clinical data (Permit No. 2018–13618).

**Figure 1:**
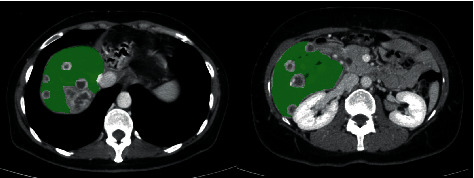
Exemplary contrast-enhanced axial CT slices in the portal venous phase depicting the volumetric measurement of tumor-free liver tissue for one patient using Aquarius iNtuition® software.

We evaluated contrast-enhanced computed tomography (CT) or magnetic resonance imaging at diagnosis and during the patients' course of disease to determine the size and number of intrahepatic lesions as well as the presence of distant metastases in the lung, the peritoneum, bones, other organs, and soft tissues other than lymph nodes. At the time of first development of distant metastases, hepatic tumor burden as percentage of total liver parenchyma was measured by volumetric assessment using dedicated third-party software (Aquarius iNtuition^©^; TeraRecon, Foster City, CA, USA; Figure 1) and categorized into three groups (<25%, 25–50%, >50%). Patients after resection without hepatic recurrence but distant metastases were categorized as “exclusively extrahepatic metastases” (EEM).

Occurrence and distribution of distant metastases were evaluated using the statistical software package R 3.5.1 [16]. Survival analyses were performed using the “survival” and “survminer” (https://cran.r-project.org/package=survival, https://cran.r-project.org/package=survminer, accessed on 31.12.2019) packages. In rare cases of missing laboratory values, these were imputed using the “mice” package (https://cran.r-project.org/package=mice, accessed on 31.12.2019). Log-rank tests and Kaplan–Meier curves were used for survival analysis between strata. Univariate and multivariate Cox proportional hazards regression models were fitted to determine the influence of predictors. As this analysis has exploratory intention, *P*‐values should be interpreted in a descriptive manner. *P* values < 0.05 were considered statistically significant.

## 3. Results

### 3.1. Occurrence and Location of Distant Metastases

**Figure 2:**
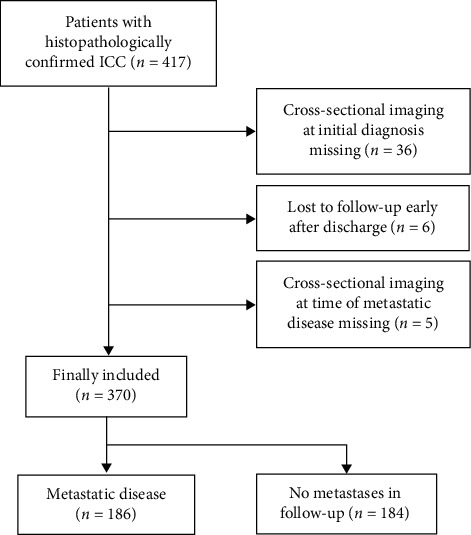
STROBE flowchart showing the number and the reasons for dropout.

Of the 417 patients, 47 had to be excluded for reasons as described in the STROBE flowchart (Figure 2); the remaining 370 patients were included in this study. A total of 200/370 patients underwent primary surgical resection, while 170/370 patients were considered nonresectable by an interdisciplinary board due to their general state of health or advanced tumor stage asserted by imaging studies or surgical exploration. Of the 200 patients undergoing resection, 7 had distant metastases at initial diagnosis, while 68 developed distant metastases during follow-up. Of the 170 nonresectable patients, 52 initially presented with distant metastases and 59 developed distant metastases during the course of disease. Further baseline characteristics of the patients at the time of the first development of distant metastases are depicted in Table 1.

**Figure 3:**
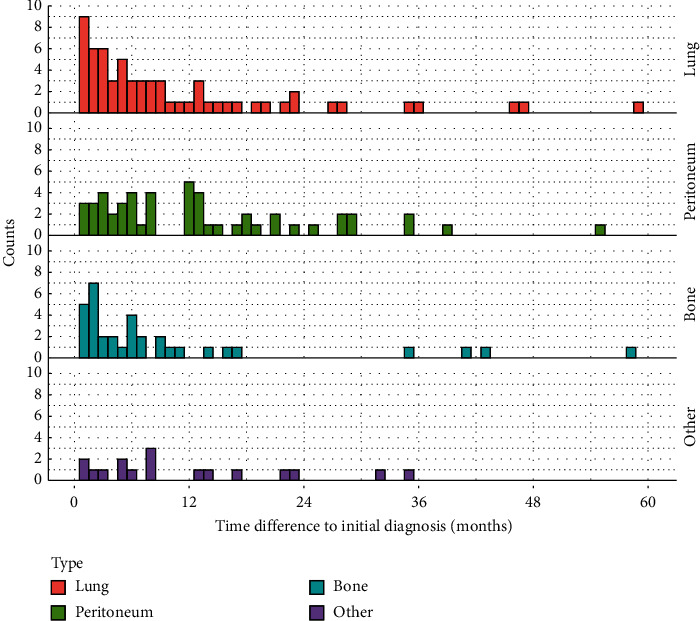
Histogram depicting time differences between initial diagnosis and metastatic occurrence for each site.

The most common metastatic site was the lung (*n* = 105, 28%), followed by the peritoneum (*n* = 81, 22%) and bones (*n* = 50, 14%). Other metastatic sites included the adrenals (*n* = 8, 2%), brain (*n* = 3, 1%), spleen (*n* = 5, 1%), and soft tissues/skin (cutaneous metastases) (*n* = 8, 2%); these sites are henceforth referred to as “other.” The time distribution of metastatic occurrence for each site is depicted in Figure 3.

### 3.2. Survival of Patients with Distant Metastases

**Figure 4:**
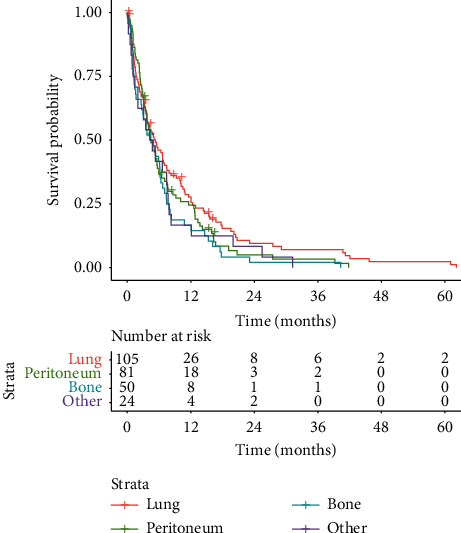
Kaplan–Meier curves of OS stratified according to metastatic sites.

When investigating survival from the time of detection of metastatic spread until death or loss to follow-up, all metastatic sites showed similar survival curves without any statistically significant difference. After detection of lung metastases, the median OS was 5.3 months; after peritoneal metastases, it was 4.5 months; and after bone metastases, it was 4.4 months. Pooled patients with metastases other than the three aforementioned locations showed an OS of 4.5 months (Figure 4). Pairwise comparisons between survival times using the log-rank test resulted in *P* values ranging from 0.29 to 0.75, with the lowest *P* value of 0.29 between patients with lung metastases and those with bone metastases.

**Figure 5:**
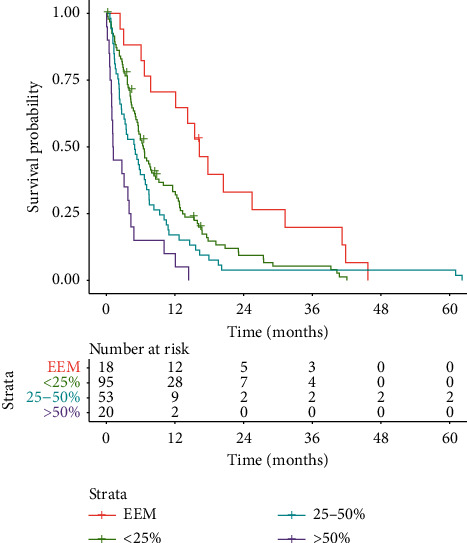
Kaplan–Meier curves of OS stratified according to hepatic tumor burden at the time of first metastatic occurrence (EEM, exclusively extrahepatic metastases).

However, when stratifying patients according to hepatic tumor burden at the time of first metastatic occurrence, survival was significantly different. The residual survival of formerly resected patients with exclusively extrahepatic metastases was 16.3 months compared with 6.5 months, 4.9 months, and 1.2 months for patients with <25%, 25–50%, and >50% hepatic tumor burden, respectively (Figure 5). Pairwise comparisons among survival times resulted in *P* values < 0.01 for all combinations except comparing the “<25%” with the “25–50%” group, where the *P* value was 0.12.

Kaplan–Meier curves of patients with metastatic disease at initial diagnosis compared with patients without metastatic spread at initial diagnosis can be found in Supplementary Materials (Figure S1, median OS 4.2 months vs. 17.7 months, *P* < 0.001). Moreover, survival analysis was performed comparing patients with metastatic disease at the time of tumor recurrence with patients without metastatic disease at the time of recurrence (Figure S2, median residual OS 6.4 months vs. 20.6 months, *P*=0.01).

**Figure 6:**
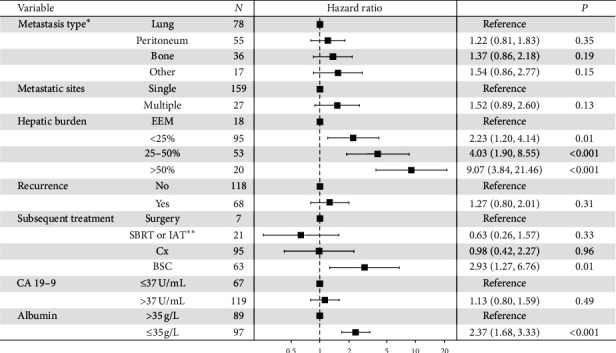
Multivariate Cox hazard regression for survival from the time of first metastatic occurrence (EEM, exclusively extrahepatic metastases; SBRT, stereotactic body radiotherapy; IAT, intra-arterial therapy; Cx, chemotherapy; BSC, best supportive care; CA 19-9, carbohydrate antigen 19-9). ^*∗*^In case of simultaneous metastatic spread to two or more different sites at the time of first metastatic occurrence, the more rarely observed metastasis was counted. ^*∗∗*^In combination with or without chemotherapy.

In multivariate Cox hazard regression, hepatic tumor burden, liver function, and subsequent treatment were significant predictors of survival (Figure 6). While the categories of “bone” and “other” metastases showed an increased hazard, the effect was not significant. Hepatic tumor burden was the factor with the highest hazard ratios (HRs) in multivariate analysis.

## 4. Discussion

In our cohort, the number of patients with extrahepatic metastases was high with every second patient developing extrahepatic metastases during the course of disease. Distant metastases were associated with poor outcome, irrespective of the metastatic site. The rarer metastatic locations showed an increased hazard; however, the effect was not significant. In contrast, the amount of hepatic tumor burden and the patient's ability to tolerate further treatment were the strongest predictors of survival in multivariate Cox hazard regression analysis.

Literature on the investigated topic is scarce, particularly regarding the longitudinal investigation of metastatic occurrence and different sites of metastatic spread. The SEER-based study by Wu et al. [15] investigated patterns of distant extrahepatic metastases at initial diagnosis in both HCC and ICC. However, they only present pooled survival analyses for both cancer entities. Moreover, while the authors claim in their conclusion that there were profound differences in risk of mortality among distant extrahepatic metastatic sites, the presented confidence intervals of HRs overlap for lung, bone, and distant lymph node metastases, with only the HR of brain metastases significantly differing from the other metastatic sites.

In both the study by Wu et al. [15] and the current study, lung metastases were the most common extrahepatic manifestation. However, in their study, the second most common metastases were bone metastases. It is unclear whether peritoneal metastases, the second most common metastases in our study, were investigated. Frega et al. [17] investigated the occurrence of brain metastases from biliary tract cancer in 450 patients and found an incidence of about 1.4%, with a median OS from detection of brain metastases of 4 months. This corresponds with the subset of patients developing brain metastases in our cohort. Older, autopsy-based studies featured only small patient numbers not exceeding 50 patients, and the share of patients affected by distant metastases differs considerably among studies. Lung metastases have been reported in 20–40%, and bone metastases in 10–55% of patients [18–20]. The results presented in our study fall within these broad ranges.

Even though the survival of patients with ICC was generally poor once metastases were detected, there was no discernible influence of metastatic sites on survival in our cohort. This corresponds with clinical routine in that liver failure, and especially, biliary complications can pose life-threatening situations for patients with advanced ICC [21], whereas, for example, respiratory failure due to lung metastases is a rare event that has only been described in case reports [22]. The number of patients who developed brain metastases in our study was very small (*n* = 3), and the aggregated tumor location “other” did not show a significantly worse outcome compared with the three main sites.

Hepatic tumor burden was the strongest predictor of poor survival in our study. Formerly resected patients with exclusively extrahepatic metastases showed the best prognosis of 16.3 months, whereas patients with more than 50% hepatic tumor infestation had an abysmal mean OS of only 1.2 months (*P* < 0.001). In previous studies investigating ICC, multifocality and tumor size were established as risk factors [12, 23]. In other tumor entities, a tumor burden score based on tumor size and number was also recently proposed for risk stratification for patients with colorectal liver metastasis, demonstrating good prognostic discriminatory power in internal and external validation [24].

In addition to hepatic tumor burden, low serum albumin was also associated with increased mortality in our study. While the mechanism of protein synthesis and distribution is complex, albumin levels have been associated with liver function and patient survival in critically ill patients and cancer patients [25–27]. Recently, high serum albumin was also found to confer a survival advantage in patients with hilar cholangiocarcinoma [28].

Regarding subsequent treatment, it is important to note that this factor is influenced by a multitude of factors including performance status, liver function, and previous treatments. Thus, a selection bias is inherent, and it is not surprising that patients who received merely best supportive care once metastases were detected showed the worst prognosis compared with other treatment groups [29]. However, it is important to stress that hepatic tumor burden maintained its role as the strongest predictor of survival in multivariate Cox regression even when subsequent treatment was included as a cofactor.

Systemic chemotherapy is the mainstay of palliative treatment [7]; however, intra-arterial therapies are considered on an individual basis. While single-institution studies for TACE and SIRT are promising [30, 31], systematic reviews stress the heterogeneity among studies and the variance in patient selection and indications [32]. Prospective randomized controlled trials comparing intra-arterial therapy with systemic treatment are still ongoing (e.g., SIRCCA trial, ClinicalTrials.gov Identifier: NCT02807181).

A small number of patients underwent surgical treatment despite the presence of distant metastases (*n* = 7). In most of these cases, liver resection was performed despite the presence of small pulmonary nodules, which were later confirmed to be metastases in follow-up imaging (*n* = 4). Furthermore, liver resection was performed once in the knowledge of a bone metastasis (*n* = 1). Two patients underwent surgery to excise a peritoneal metastasis (*n* = 1) and a cutaneous metastasis (*n* = 1) respectively. However, in nonmetastasized patients, there has been emerging evidence supporting re-resection for recurrent ICC [33]. Moreover, Zhang et al. [34] reported a survival benefit for Asian patients with metastasized hepatolithiasis-associated ICC undergoing palliative resection. Yamada et al. [35] published a case report of long-term survival after surgical resection for recurrent hepatic and pulmonary metastases of ICC. Therefore, there might be room for advocating liver resection when the extrahepatic manifestations are limited.

Our analysis had several limitations. First and foremost, the study was single-centered and conducted in a retrospective fashion. Therefore, a bias due to center-specific treatment may be present. Independent external validation of the results presented in this study is still missing, and future multicenter approaches may be necessary to validate our results. Second, the number of investigated patients (*n* = 370) was moderate. However, given the low incidence of ICC in Western countries, existing studies investigating patients with ICC have similar or even considerably smaller sample sizes. Due to the long study period, both improved imaging technologies and improved treatment options over time carry a bias that is difficult to control for. Availability and quality of cross-sectional imaging have improved over the years, with more stringent follow-ups towards the end of the recruitment period. Therefore, a selections bias towards the later years in the recruitment period is present and patients with metastatic disease might have been missed in earlier years. Furthermore, patients received different chemotherapy regimen over time; the current standard of gemcitabine and cisplatin was introduced in 2010 following the UK-ABC2 trial [7], and outcomes might have improved since then due to adherence to this regimen. Moreover, there were no stipulated treatment options once metastases were detected, but therapeutic concepts were devised in interdisciplinary tumor boards for each patient on an individual basis.

## 5. Conclusions

While the presence of distant metastases is frequent in patients with ICC and is associated with a poor outcome, there was no discernible difference in OS between metastatic sites. However, hepatic tumor burden was the life-limiting risk factor in a majority of patients. Therefore, in addition to chemotherapy, interdisciplinary approaches including resection and intra-arterial therapy might be considerations on an individual basis to achieve hepatic tumor control even in the presence of distant metastases if deemed oncologically reasonable.

## Figures and Tables

**Table 1:** Baseline characteristics of patients with distant metastases.

		All (*n* = 186)	Initially resected (*n* = 75)	Initially nonresectable (*n* = 111)
Age, years, median (IQR)		63.8 (56–72)	61.3 (55–68)	67.0 (57–74)
Sex, *n* (%)	Male	112 (60.2)	43 (57.3)	69 (62.2)
	Female	74 (39.8)	32 (42.7)	42 (37.8)
Initial first-line therapy, *n* (%)	Best supportive care	21 (11.3)	0 (0.0)	21 (18.9)
	Chemotherapy	62 (33.3)	0 (0.0)	62 (55.9)
	SBRT or IAT with or w/o chemotherapy	28 (15.1)	0 (0.0)	28 (25.2)
	Surgery	75 (40.3)	75 (100.0)	0 (0.0)
Type of distant metastasis, *n* (%)^†^	Lung	105 (56.5)	40 (53.3)	65 (58.6)
	Peritoneum	81 (43.5)	30 (40.0)	51 (45.9)
	Bone	50 (26.9)	18 (24.0)	32 (28.8)
	Other^‡^	24 (12.9)	12 (16.0)	12 (10.8)
Multiple affected metastatic sites, *n* (%)	At first metastatic occurrence	27 (14.5)	6 (8.0)	21 (18.9)
	Over the disease course	58 (31.2)	19 (25.3)	39 (35.1)
Sum of intrahepatic lesions^§^, mm, median (IQR)		103 [47–167]	42 [8–101]	150 [86–191]
Hepatic tumor burden^§^, *n* (%)	EEM	18 (9.7)	18 (24.0)	0 (0.0)
	<25%	95 (51.1)	47 (62.7)	48 (43.3)
	25–50%	53 (28.5)	9 (12.0)	44 (39.6)
	>50%	20 (10.7)	1 (1.3)	19 (17.1)
CA 19-9 serum levels^§^, U/mL, median (IQR)		107 [22–1442]	37 [16–261]	137 [32–1862]
Albumin^§^, g/L, median (IQR)		35 [31–39]	36 [33–40]	34 [29–39]
Subsequent therapy^§^, *n* (%)	Best supportive care	63 (33.9)	17 (22.7)	46 (41.5)
	Chemotherapy	95 (51.1)	48 (64.0)	47 (42.3)
	SBRT or IAT with or w/o chemotherapy	21 (11.3)	3 (4.0)	18 (16.2)
	Surgery	7 (3.7)	7 (9.3)	0

IQR, interquartile range; EEM, exclusively extrahepatic metastases; CA 19-9, carbohydrate antigen 19-9; SBRT, stereotactic body radiotherapy; IAT, intra-arterial therapy. ^†^The sum of distant metastases is >100% because patients could have more than one metastatic site. ^‡^Other sites include the brain, adrenals, spleen, and soft tissues/skin (cutaneous metastases). ^§^At time of first metastatic occurrence.

## Data Availability

The data used to support the findings of this study are available from the corresponding author upon request.

## Abbreviations

ICC: Intrahepatic cholangiocarcinoma
HCC: Hepatocellular carcinoma
TACE: Transarterial chemoembolization
SIRT: Selective internal radiation therapy
SEER: Surveillance, Epidemiology, and End Results
OS: Overall survival
CT: Computed tomography
EEM: Exclusively extrahepatic metastases
HR: Hazard ratio.

## Supplementary Materials

Supplementary Materials Figure S1: Kaplan–Meier curves of OS stratified according to presence of distant metastases at initial diagnosis. Figure S2: Kaplan–Meier curves of residual OS at time of recurrence stratified according to presence of distant metastases.
