# Supplementary material for: Distant Metastases in Patients with Intrahepatic Cholangiocarcinoma: Does Location Matter? A Retrospective Analysis of 370 Patients
Source: J Oncol. 2020 Oct 10;2020:7195373. doi: 10.1155/2020/7195373 (PMC7569461; doi:10.1155/2020/7195373)
Supplement: Supplementary Materials — Figure S1: Kaplan–Meier curves of OS stratified according to presence of distant metastases at initial diagnosis. Figure S2: Kaplan–Meier curves of residual OS at time of recurrence stratified according to presence of distant metastases. [file 7195373.f1.doc]

**Supplement**

**
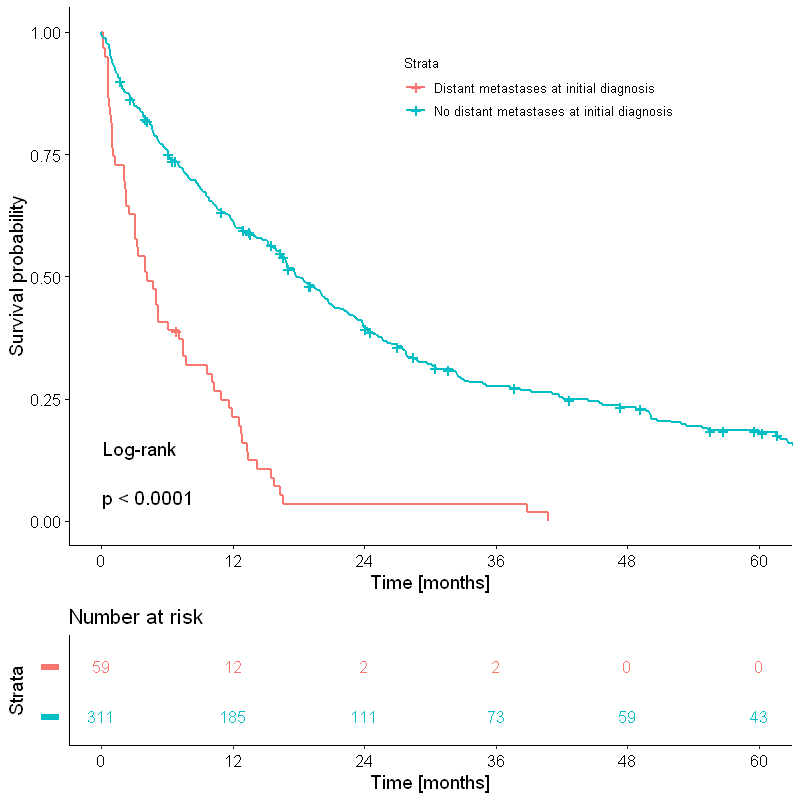
**

Figure S1 Kaplan-Meier curves of OS stratified according to presence of distant metastases at initial diagnosis.


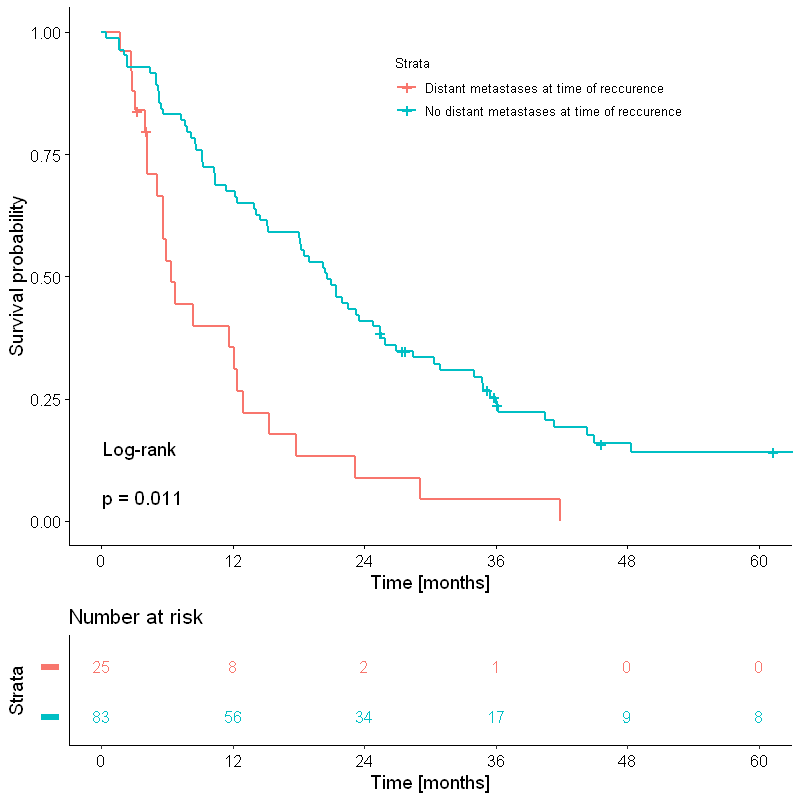


Figure S2 Kaplan-Meier curves of residual OS at time of recurrence stratified according to presence of distant metastases.
